# Structure–Function Relationship of Organic Semiconductors: Detailed Insights From Time-Resolved EPR Spectroscopy

**DOI:** 10.3389/fchem.2019.00010

**Published:** 2019-02-01

**Authors:** Till Biskup

**Affiliations:** Institute of Physical Chemistry, University of Freiburg, Freiburg, Germany

**Keywords:** organic semiconductors, EPR (electron paramagnetic resonance), triplet state, structure—function relationship, electronic structure, morphology

## Abstract

Organic photovoltaics (OPV) is a promising technology to account for the increasing demand for energy in form of electricity. Whereas the last decades have seen tremendous progress in the field witnessed by the steady increase in efficiency of OPV devices, we still lack proper understanding of fundamental aspects of light-energy conversion, demanding for systematic investigation on a fundamental level. A detailed understanding of the electronic structure of semiconducting polymers and their building blocks is essential to develop efficient materials for organic electronics. Illuminating conjugated polymers not only leads to excited states, but sheds light on some of the most important aspects of device efficiency in organic electronics as well. The interplay between electronic structure, morphology, flexibility, and local ordering, while at the heart of structure—function relationship of organic electronic materials, is still barely understood. (Time-resolved) electron paramagnetic resonance (EPR) spectroscopy is particularly suited to address these questions, allowing one to directly detect paramagnetic states and to reveal their spin-multiplicity, besides its clearly superior spectral resolution compared to optical methods. This article aims at giving a non-specialist audience an overview of what EPR spectroscopy and particularly its time-resolved variant (TREPR) can contribute to unraveling aspects of structure–function relationship in organic semiconductors.

## 1. Introduction

Using organic photovoltaics (OPV) to account for the increasing demand for energy in form of electricity becomes more and more important (Darling and You, 2013; Yeh and Yeh, 2013; Wang et al., 2016; Haque et al., 2018; Xue et al., 2018). Undoubtedly, there is large progress in the field witnessed by the steady increase in efficiency of OPV devices (Green et al., 2018). Replacing conventional inorganic (silicon-based) semiconductors with organic molecules comes with a number of advantages, such as mechanical flexibility (Li et al., 2017), low cost (Forrest, 2004; Mulligan et al., 2015; Gambhir et al., 2016), and probably most important, the nearly infinite possibilities of tailoring molecules by means of organic synthesis for each special application (Guo et al., 2013; Liu et al., 2015; Müllen and Pisula, 2015). Nevertheless, we still lack proper understanding of some of the core aspects of light-energy conversion, demanding for systematic investigation on a fundamental level.

Illuminating conjugated polymers used in OPV devices not only leads to excited states, but sheds light on some of the most important aspects of device efficiency in OPVs as well. The interplay between electronic structure, morphology, flexibility, and local ordering, while at the heart of structure—function relationship of organic electronic materials, is still barely understood. Electron paramagnetic resonance (EPR) spectroscopy is perfectly suited to address these issues on a molecular scale, as most species formed in the course of charge generation and charge separation in organic solar cells are inherently paramagnetic. Time-resolved EPR (TREPR) spectroscopy, in particular, is a powerful tool to characterize the various short-lived excited species that are created after light excitation of organic molecules. The big advantage of EPR spectroscopy over more conventional, optical spectroscopy is its molecular resolution due to its inherent sensitivity to the local environment of the electron spin used as a probe for the electronic structure of the molecule. Optical spectroscopy, on the other hand, provides a clearly superior time resolution as compared to EPR spectroscopy, due to inherent physical constraints of the latter. Additionally, optical spectroscopy is much more sensitive compared to EPR spectroscopy. This is due to the difference between the energy levels involved in the transitions detected, resulting in much higher population differences of these energy levels in case of optical spectroscopy.

Whereas the focus of the author's research is on applying TREPR spectroscopy to short-lived excited states, predominantly triplet excitons, EPR spectroscopy can contribute even further to a more thorough understanding of organic electronic materials (Niklas and Poluektov, 2017). Hence, after a primer on EPR spectroscopy, this article first gives an overview of paramagnetic states in OPV devices and organic semiconductors, followed by a more detailed description of the available EPR-spectroscopic experiments to characterize each of these paramagnetic species. Afterwards, the relevant characteristics of triplet excitons directly observed by TREPR spectroscopy are detailed a bit more. Finally, in a series of showcase studies, TREPR spectroscopy of triplet excitons is demonstrated to reveal information on different aspects of the all-important structure—function relationship of organic semiconductors, such as solution and film morphology, conformational flexibility, local ordering, triplet routes, and electronic structure. For aspects of charge separation and charge transport and the relevant contributions of EPR spectroscopy that are not covered here, the reader is referred to the literature (Kraffert and Behrends, 2017; Niklas and Poluektov, 2017).

## 2. A Primer on EPR Spectroscopy

As not everybody in the field of organic electronics might be familiar with EPR spectroscopy, a very brief introduction will be given. For further details, the interested reader is referred to the literature (Carrington and McLachlan, 1967; Atherton, 1993; Weil and Bolton, 2007; Brustolon and Giamello, 2009; Goldfarb and Stoll, 2018). EPR spectroscopy and its more widely used relative nuclear magnetic resonance (NMR) spectroscopy are both based on the same fundamental theory, namely the interaction of spins with magnetic fields (Abragam, 1961; Slichter, 1963; Poole and Farach, 1987). Whereas NMR spectroscopy deals with the interaction of nuclear spins with external magnetic fields and is perhaps the most valuable single analytic technique available to the chemist, the subject of EPR spectroscopy is the interaction of electron spins with external magnetic fields in their surrounding. Generally, interactions of the electron spin with its environment can be distinguished according to their respective origin. The two most commonly encountered interactions are the electron Zeeman interaction of an electron spin with the external magnetic field, described by the Hamilton operator $H_{\text{EZ}}$, and the hyperfine interaction between the electron spin and the spins of surrounding nuclei, described by the Hamiltonian $H_{\text{HF}}$. The nuclear Zeeman interaction of the nuclear spins with the external magnetic field, described by the Hamiltonian $H_{\text{NZ}}$, is usually quite weak and often neglected. In systems with more than one unpaired electron spin and those electron spins interacting with each other, two additional interactions come into play, namely the dipolar interaction described by the term $H_{\text{ZFS}}$ (frequently alternatively named $H_{\text{DD}}$ in the literature) whose effect is often referred to as zero-field splitting (ZFS) due to its independence from an externally applied field, and the exchange interaction described by the term $H_{\text{EX}}$. The dipolar coupling described by $H_{\text{ZFS}}$ consists of two contributions, namely a spin–orbit term and a spin–spin term. The former can usually be safely ignored for organic molecules, as the spin–orbit coupling depends on the atomic charge in at least the fourth power. The same argument holds for explaining why the anisotropy of the electron Zeeman interaction, described by the **g** tensor, is usually rather small. This is why often, particularly for organic triplet states, instead of a tensor, an isotropic *g* value close to that of the free electron, *g*_e_ ≈ 2.002319, gets used for spectral simulations. Summing up all relevant interactions yields the total electron spin Hamilton operator H of a system:

(1) $$\begin{array}{r} H=H_{\text{EZ}}+H_{\text{HF}}+H_{\text{NZ}}+H_{\text{ZFS}}+H_{\text{EX}}. \end{array}$$

Depending on the system investigated, one or several of these terms can be omitted due to their contribution to the overall energy of the system being too minor or nonexisting in case of electron spins not interacting with each other. One such system described later in more detail are triplet states of organic molecules where only the electron Zeeman and the dipolar interaction are relevant for describing the spectra. Here, the hyperfine interaction is considered only a minor perturbation and hidden in the (inhomogeneous) line broadening.

Although pulsed detection schemes have been developed for EPR spectroscopy in the last decades, because of the much shorter spin relaxation times as compared to NMR spectroscopy, continuous-wave (cw) detection schemes are still widespread and remain highly important (van der Est, 2016). Due to the very small difference between the energy levels of an electron spin system, usually resonating structures and in case of cw detection lock-in amplifiers are used to increase the signal-to-noise ratio. The latter leads to characteristic derivative line shapes of the absorption lines, whereas in pulsed detection schemes, the absorptive line shape is retained. Recording EPR spectra in presence of a continuous microwave field, as in cw-EPR spectroscopy, results in a much narrower excitation as compared to pulsed detection schemes. Therefore, spectra recorded using either cw or pulsed techniques usually have different respective line widths. One major reason for using pulsed detection is its versatility, allowing to selectively probe different interactions with high precision, depending on the pulse sequence used. For an excellent introduction into pulsed EPR spectroscopy, the reader is referred to Schweiger and Jeschke (1991). A good overview of different EPR techniques used to investigate light-induced paramagnetic species in organic photovoltaic devices, with a particular focus on charge-transfer states and polarons, can be found in Niklas and Poluektov (2017).

A minor modification of conventional cw and pulsed EPR spectroscopy is light-induced EPR (LEPR) spectroscopy using cw illumination to create paramagnetic species in a sample, e.g., polarons in a blend of donor and acceptor material as used in organic photovoltaics (Dyakonov et al., 1999). As the paramagnetic species are in steady state on the time scale of the EPR experiment, this allows for investigating them using all the available different EPR-spectroscopic techniques, including advanced pulsed and double-resonance methods. Although light-induced, these paramagnetic species are still thermally populated with respect to their energy levels, resulting in purely absorptive signals that may appear in derivative line shape, depending on the detection scheme used.

**Figure 1:**
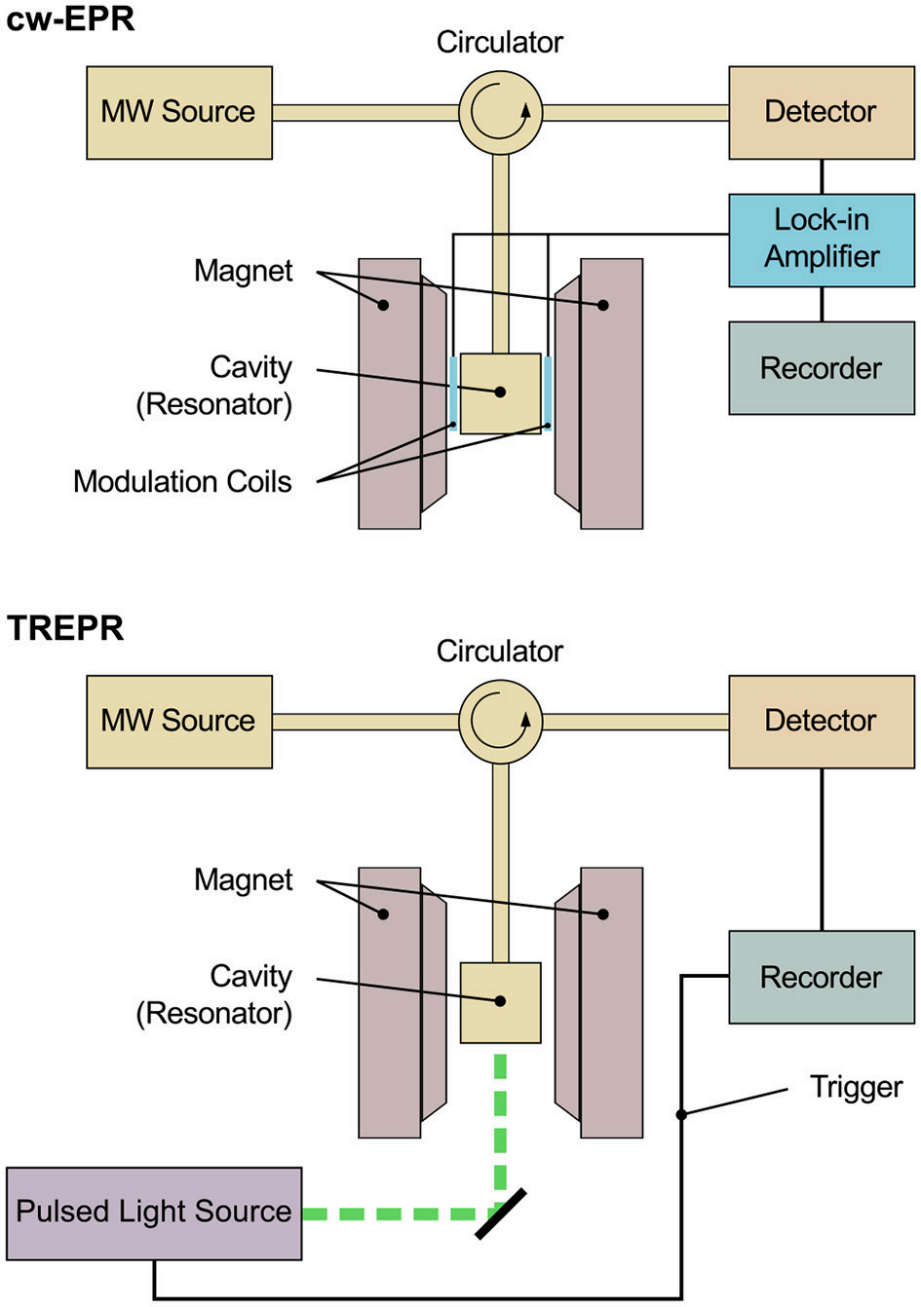
Schematic diagram of an EPR spectrometer for conventional continuous-wave and time-resolved measurements. The basic setup is identical in both cases. The sample is placed in a cavity (resonator) situated between the poles of the magnet. Microwave is fed into the cavity from a microwave source (MW source), and the microwave reflected is directed toward the detector by means of a circulator. For cw-EPR spectroscopy, a lock-in detection scheme is used for enhanced signal-to-noise ratio, comprising of additional modulation coils placed on the inside of the poles of the magnet, modulating the external magnetic field. The signal is detected phase-sensitive with respect to this external modulation. Typical modulation frequencies are in the range of 10–100 kHz, thus restricting the available time resolution technically to several tens of microseconds, whereas in practice, most spectrometers only allow a lower limit of about a millisecond. Due to the non-Boltzmann population of the energy levels of spin-polarized paramagnetic states created by light excitation through a pulsed light source, signals can be detected in a direct manner in TREPR spectroscopy, meaning excluding lock-in detection. This allows for a much higher time resolution down to a few nanoseconds.

Investigating transient paramagnetic species usually created using a short (typically nanosecond) light pulse, as in TREPR spectroscopy, leads to an entirely different situation. While steady-state paramagnetic species under continuous illumination (LEPR) exhibit Boltzmann population of their respective energy levels, due to their transient nature, the energy levels of the paramagnetic species created with short light pulses are far from the thermal equilibrium. Usually termed spin polarization, this phenomenon gives rise to a large signal enhancement of the corresponding TREPR spectra that show signals in both, enhanced absorption (A) and emission (E). This signal enhancement allows to omit the lock-in detection scheme used in conventional cw-EPR spectroscopy and therefore to increase time resolution up to about 10 ns. The physical limit of the time resolution is given by the precession frequency of the magnetization vector about the external magnetic field directly related to the frequency of the incident microwave. Technically and practically, however, the slowest component in the signal path determines the actual time resolution of a spectrometer, be it the bandwidth of the detection or the resonator. Additionally, increasing the time resolution always comes to the price of a decreased signal-to-noise ratio. Note that with using lock-in detection and a modulation frequency of typically 100 kHz, technically speaking, the available time resolution would be in the order of several tens of microseconds, whereas in practice, most spectrometers only allow a lower limit of about a millisecond. This renders lock-in detection schemes very slow and mostly not applicable to short-lived light-induced paramagnetic species. Figure 1 presents schematic diagrams of an EPR spectrometer both, for steady-state as well as time-resolved measurements. Whereas the basic setup is identical, for cw-EPR, a lock-in detection scheme is used to enhance the signal-to-noise ratio. Due to the non-Boltzmann population of the energy levels of spin-polarized paramagnetic states created by light-excitation through a pulsed light source, TREPR signals can be detected in a direct manner, resulting in the comparably high time resolution mentioned. Details of the respective setup can be found in the literature for cw-EPR (van der Est, 2016) and TREPR (Weber, 2017) spectrometers, respectively. Note that pulsed detection schemes can be used as well to investigate transient spin-polarized paramagnetic species. The rather small repetition rate of conventional pulsed lasers used for excitation as compared to the pulsed detection without additional optical illumination, however, together with the unavoidable sample degradation due to prolonged excitation sometimes limits this approach. Whereas techniques for background correction are available, generally, pulsed detection will only record the EPR signal at a fixed delay time after the laser pulse. Therefore, obtaining the complete time profile after the laser pulse would require repeated illumination in pulsed detection mode.

## 3. Paramagnetic States in Organic Photovoltaic Devices

Most species formed in the course of charge generation and charge separation in organic solar cells are inherently paramagnetic. Nevertheless, the role of the electron spin on kinetics of recombination and therefore eventually the efficiency of OPV devices has only recently been highlighted (Rao et al., 2013; Wohlgenannt et al., 2015). In the dark, however, ideally there should be no paramagnetic species present. In any case, it is important to distinguish between transient, light-induced paramagnetic states and stable species present in the dark as well. The latter may or may not originate from light-induced degradation processes, but they persist in the device in contrast to the former. As light-induced transient paramagnetic states are intrinsic to the functioning of OPV devices, they are first introduced in some detail, together with an overview of the different EPR-spectroscopic techniques used to characterize them. Afterwards, the same is done for the stable paramagnetic states in those devices.

### 3.1. Transient Paramagnetic States After Light Excitation

**Figure 2:**
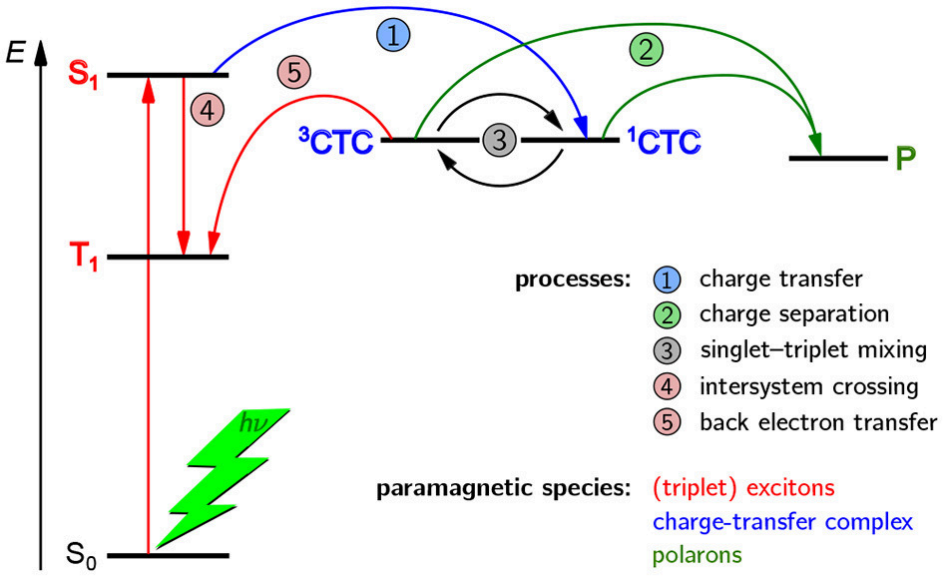
Possible paramagnetic states in OPV devices formed after light excitation and their origins. In principle, three kinds of paramagnetic states can occur in OPV devices, namely charge-transfer complexes (CTC) consisting of coulombically coupled interacting radicals, separated charge carriers (polarons, P), and triplet-configured excited states (triplet excitons). In an ideal device with very fast charge separation (process 2), only the polarons would be detectable. However, longer-lived CTCs and triplet excitons (^3^T) are often encountered and give rise to characteristic signatures in TREPR spectroscopy. Note that all three kinds of paramagnetic states give rise to characteristic signals in EPR spectroscopy and can be clearly distinguished from each other. For details, see the text.

A schematic overview of the paramagnetic states possibly been formed upon light excitation in an organic solar cell is given in Figure 2. If the organic semiconductor, here the donor, absorbs a photon of the appropriate energy, it gets excited into an excited singlet state. In an idealized picture, the excited state migrates toward the interface with the acceptor phase and forms there a coulombically coupled charge-transfer state, often termed charge-transfer complex (CTC), upon transferring a charge onto the acceptor. This CTC is split in due course to give free charge carriers (polarons) that migrate toward the electrodes with opposite polarity where they are collected, hence giving rise to a current. As both, positive and negative charge carriers bear an unpaired electron, they are intrinsically paramagnetic and therefore accessible by EPR spectroscopy under continuous illumination (LEPR). In a real device, a number of other, competing processes can take place as well. If charge separation is slower, the CTC can undergo singlet–triplet mixing, making recombination to an energetically lower-lying triplet state by back electron transfer possible. Excited states might as well directly undergo spin-forbidden intersystem crossing to a triplet state. Both, CTCs and triplet excitons, are as well intrinsically paramagnetic and hence accessible by EPR-spectroscopic methods. Due to these states being rather short-lived, usually on the order of a few microseconds, detection needs to be rather fast, typically following pulsed laser excitation and using TREPR spectroscopy.

From this first overview it is obvious that three different kinds of paramagnetic states can generally occur upon light excitation of OPV devices, namely polarons, CTCs and triplet excitons. In an ideal device with very fast charge separation, only the polarons would be detectable, by means of LEPR spectroscopy. However, longer-lived CTCs and triplet excitons are often encountered and give rise to characteristic signatures in TREPR spectroscopy. An important aspect to note is that EPR spectroscopy can unequivocally differentiate between these three kinds of paramagnetic species, as well as other, less frequently occuring states with higher spin multiplicity.

#### 3.1.1. Polarons

From an EPR spectroscopist's perspective, positive and negative polarons (charge carriers) are radicals, i.e., spin-1/2 particles or doublet states, that can be used as a probe for their molecular environment. Due to their transient nature, they are normally probed using LEPR with continuous illumination, resulting in a steady-state concentration of polarons sufficient to yield reasonable EPR signals. *In situ* illumination can be achieved using either white or monochromatic light from lamps, diodes or even continuous-wave (cw) lasers. Besides the continuous illumination that needs not to be synchronized in any way with the spectrometer, a standard EPR setup can be used with either cw or pulsed EPR detection, opening up the possibility to perform all kinds of experiments, even advanced pulse EPR experiments. The only limitation of LEPR spectroscopy is the need for investigating blends of donor and acceptor materials, even if one is only interested in the charge carriers contained in one of them, as otherwise, due to the low dielectric constant of organic semiconductors, no charge separation will occur and no polarons will be formed. Due to the small contribution of spin–orbit coupling, organic molecules normally have comparably isotropic *g* tensors with values close to the *g* value of the free electron, *g*_e_ ≈ 2.002319. Hence, the signals of both types of polarons usually strongly overlap, at least at conventional microwave frequencies and magnetic fields (X-band: ≈9.5 GHz microwave frequency, 340 mT external magnetic field), making interpretation of the resulting signals and disentanglement of the spectral components rather advanced. This is one reason for using PC_61_BM rather than PC_71_BM for EPR investigations, despite the normally higher efficiency of the latter. The smaller *g*_iso_ value of PC_61_BM as compared to most organic radicals leads to a separation of the spectral components (Krinichnyi and Yudanova, 2012). Other approaches would be to use high-field EPR (Poluektov et al., 2010; Niklas et al., 2013), or, if even this is not sufficient due to overlapping *g* tensor components, advanced EPR methods relying on either different relaxation times for the different polaron types or other characteristics (Van Landeghem et al., 2018).

A somewhat special case of probing polarons is to investigate chemically doped molecules resembling the polarons present upon illumination (Aguirre et al., 2008). The advantage over blends and using LEPR is obvious: here, only one species is present, rendering any approach to disentangle the resulting spectra obsolete. Ideally, the chemically induced species resembles the light-induced one. This is, however, not necessarily the case, as the presence of counter ions, chemical modifications, or overdoping can lead to differences between chemically and light-induced species. Different EPR-spectroscopic techniques are sensitive to a different degree to these effects, depending on the specific interactions probed.

#### 3.1.2. Charge-Transfer Complexes (CTCs)

Another light-induced and potentially paramagnetic species present in OPV devices under operating conditions are CTCs, i.e., coulombically bound charge pairs residing at the interface between donor and acceptor phase. Other names include spin-correlated polaron pairs (SCPP), (spin-correlated) radical pairs (SCRP), and CT states (Kobori et al., 2013; Miura et al., 2014, 2016; Kobori and Miura, 2015; Kraffert and Behrends, 2017). SCRPs have been well-known for decades from biological systems, most prominently photosynthetic reaction centers (van der Est, 2001; Bittl and Weber, 2005) and the underlying theory to describe the resulting EPR spectra has been developed accordingly (Thurnauer and Norris, 1980; Buckley et al., 1987; Closs et al., 1987; Hore et al., 1987; Stehlik et al., 1989a; Norris et al., 1990). But these paramagnetic states are far more widespread, both in biological (Weber, 2005; Biskup, 2013) and chemical systems (Wasielewski, 1992, 2006; Turro et al., 2000).

Due to their short lifetime, CTCs are usually probed using TREPR spectroscopy following pulsed laser excitation. For a good introduction into TREPR spectroscopy, see the reviews by Forbes et al. (2014) and Weber (2017). The intrinsic time resolution of a TREPR experiment is eventually limited by physical considerations, i.e., the precession frequency of the electron spin around the magnetic field. This means that the maximum achievable time resolution using conventional X-band microwave frequencies (about 10 GHz) would be in the order of a nanosecond. Therefore, TREPR is “blind” for the fast photophysical processes occurring normally on (sub)picosecond time scales (Turro et al., 2010). Additionally, one should mention that bandwidth normally comes to the cost of increased noise, hence the maximum possible time resolution of 1–10 ns is only accessible with samples exhibiting rather strong signals. Pulsed EPR methods cannot come to rescue here, as they usually don't allow for dead-time-free detection.

The characteristics of CTCs in EPR spectroscopy are spin-polarized spectra consisting of both, absorptive and emissive contributions due to the non-Boltzmann population of the four energy levels involved (Buckley et al., 1987; Hore, 1989). Additionally, the spectra are usually quite narrow, spanning only a few mT, and hence much narrower than triplet states. This is due to the much smaller dipolar interaction in CTCs as compared to triplet states by virtue of the much larger separation of the two spins in the former.

Already about 20 years ago, the first CT states have been detected in materials used for OPVs (Pasimeni et al., 2001a,b; Franco et al., 2005). However, there was a long-standing debate whether the SCRPs seen with TREPR spectroscopy are really relevant for OPV operation. Later it has been claimed that indeed, the CTCs observed using TREPR are relevant for OPV device operation, based on the similarity of the signal late after the laser pulse with the signature of the polarons observed using continuous illumination (Behrends et al., 2012). However, alternative interpretations for the signal evolution and the exclusively absorptive signal shape for late times after the laser pulse have been brought forward, based on selective decay of the energy levels involved in the SCRP (Kobori et al., 2013).

Unfortunately, there seems to be some confusion in the OPV literature about the nature of an SCRP and the possible EPR signals that can be obtained. In an ideal device at room temperature, CTCs are usually too short-lived to be detectable as SCRP by TREPR spectroscopy (Sariciftci et al., 1992; Gélinas et al., 2014; Bässler and Köhler, 2015). Furthermore, EPR spectroscopy intrinsically only detects the triplet character of a SCRP. Starting off from a singlet precursor state, i.e., an excited singlet state, as would be normally the case in OPV materials, in order to obtain an EPR signal, singlet-triplet mixing (process 3 in Figure 2) would first need to occur. This process is due to the different precession frequencies of the two coupled radicals by means of their (slightly) different environment, and typical mixing times are on the order of a few to a few ten nanoseconds (Kothe et al., 1991, 1994a,b; Weber et al., 1995), depending on the hyperfine couplings and differences in *g* values involved. Hence, EPR spectroscopy cannot distinguish between a singlet and a triplet radical pair, but only probe its (partial) triplet character. However, the spin multiplicity of the *precursor state* of the radical pair or CTC can generally be probed using multi-frequency EPR (Weber et al., 2010), or alternatively using optical spectroscopy involving applying an external magnetic field (Henbest et al., 2008; Huynh et al., 2018). With all other parameters constant, the signature of a singlet-born and a triplet-born SCRP would, in the simplest case, only change the sign of the polarization pattern. However, whereas singlet-born radical pairs exhibit no net polarization, this will generally be not the case for triplet-born radical pairs.

One perhaps rather general difference between SCRPs in biological systems and in OPV devices is the distribution of geometries of the radical-pair partners with respect to each other. A biological system, i.e., a protein, normally provides a highly homogeneous and defined environment in terms of geometrical relationship of the different radicals. In polymers or even blends of different organic semiconductors, in contrast, one would expect a rather large spread of different geometries. However, even for CTCs, geometric information can be extracted, pointing toward a rather highly ordered microenvironment on the interface formed by the bulk heterojunction (Kobori et al., 2013; Miura et al., 2014, 2016; Kobori and Miura, 2015).

#### 3.1.3. Triplet Excitons

Excitons can only be probed using TREPR spectrosopy if they are paramagnetic, meaning usually in their triplet state. Whereas triplet states are not the only paramagnetic excited states, they are by far the most common ones encountered in organic matter. Given that most organic semiconductors have a singlet ground state, triplet states are usually formed via intersystem crossing following optical excitation. Different ways to form triplet states in OPV devices upon light excitation are shown in Figure 2, and more details will be provided later on when dealing explicitly with revealing triplet formation pathways using TREPR spectroscopy. There is an ongoing discussion whether triplet states are detrimental for the overall device efficiency (Liedtke et al., 2011; Rao et al., 2013), a potential source to further increase efficiency (Yost et al., 2012), or rather an unavoidable by-product of light excitation without much impact (Kraffert et al., 2017; Benduhn et al., 2018). In any case, they clearly appear in both, pristine polymers and blends of donors and acceptors used in OPV devices (Köhler and Bässler, 2009). Hence, a detailed characterization of these states and analysis of their formation pathways is crucial, besides triplet states being useful for revealing further information on electronic structure and morphology of conjugated polymers, as will be detailed below. Due to their transient nature, typical lifetimes of triplet states ranging from nanoseconds to several hundred microseconds, TREPR spectroscopy is the method of choice to analyze and characterize these states.

#### 3.1.4. Paramagnetic States With Higher Spin

Besides polarons, CTCs and triplet excitons, there can exist paramagnetic states with higher spin states. Possible candidates are triplet-doublet pairs (quartet states) between a triplet state and a (stable) spin-1/2 state, most probably a defect center (Imamura et al., 1986; Blättler et al., 1990; Kobori et al., 1998). Others would be quintet states from two (strongly) interacting triplets, e.g., after singlet fission (Bayliss et al., 2014; Weiss et al., 2017). For an introduction into the general magnetic resonance theory of such states, namely the zero-field splitting (ZFS) see Telser (2017). These high-spin states should generally be distinguishable from each other and from triplet states by the frequency of their transient nutations (Astashkin and Schweiger, 1990; Isoya et al., 1990; Sato et al., 1997). Here, the term transient nutation refers to the precession of the bulk magnetization vector about the effective magnetic field in response to the abrupt application of an intense resonant field (Torrey, 1949; Atkins et al., 1974), or, in case of TREPR spectroscopy, to the sudden creation of the paramagnetic species in a state far from thermal equilibrium (Kim and Weissman, 1978; Stehlik et al., 1989b). Due to the short lifetime of these states, at least if they are created following pulsed light excitation, they are normally probed using TREPR spectroscopy.

### 3.2. Stable Paramagnetic Species

Besides the transient paramagnetic species occuring in OPV devices under operating conditions after pulsed or during continuous optical excitation, a series of stable paramagnetic species may or may not be present in these materials as well. Generally, these stable paramagnetic species can be divided into defects and purposefully doped molecules. In both cases, they are often charged molecules, and they clearly influence the properties of the active media and hence the function of the OPV devices. Charged molecules generally act as traps for both, excitons and charge carriers, and as traps are normally unavoidable in organic semiconductors, purposefully doping the material can help fill these energetically low-lying states and in turn enhance again charge carrier mobilities. (Fishchuk et al., 2002; Coehoorn, 2007)

#### 3.2.1. Defects

Paramagnetic defects in organic semiconductors are mostly the result of one of three processes: photochemical processes often involving oxygen species and leading to degradation (Bauld et al., 2012; Bonoldi et al., 2014; Rivaton et al., 2014; Aoyama et al., 2015; Chen et al., 2015), traces of catalysts left over from polymerization (Camaioni et al., 2012; Nikiforov et al., 2013), or deposition of metal electrodes on top of the active layers (Li et al., 2016). If light-induced, these species can accumulate as a result of prolonged illumination, particularly in case of pulsed laser excitation with its high energy densities. These stable paramagnetic defects can be probed by conventional cw-EPR (Frolova et al., 2015; Susarova et al., 2015) and further characterized using pulsed EPR spectroscopy, their effects on the devices probed using LEPR spectroscopy (Havlicek et al., 2018; Perthué et al., 2018).

#### 3.2.2. Purposefully Doped Molecules

Besides accidentially doping organic semiconductors, as described above, purposefully introducing paramagnetic states is a method in its own right (Salzmann and Heimel, 2015; Xiao et al., 2015). Normally, doping aims at enhancing the charge-carrier densities and charge-carrier mobilities in organic semiconductors (Walzer et al., 2007; Salzmann et al., 2016), but it has as well been used successfully to prevent recombination and therefore enhance charge separation and device efficiency (Zhang et al., 2012, 2013; Cho et al., 2015). The doping efficiency can be probed by conventional cw-EPR spectroscopy, either semiquantitatively (Shin et al., 2018) or quantitatively using spin counting (Kiefer et al., 2018). For an excellent introduction into quantitative EPR spectroscopy, its possibilities and requirements, the reader is referred to Eaton et al. (2010).

A somewhat special application of chemical doping in the context of EPR-spectroscopic investigation of organic semiconductors is its use to mimic charge carriers (polarons) occurring upon illumination. Chemical doping, in contrast to illumination, is applicable to pristine polymers, not only blends, and it circumvents the necessity to provide light access to the EPR spectrometer, particularly in case of high-field EPR, where this can become technically demanding. Detailed investigations of chemically doped polymers using advanced pulsed EPR methods have been successfully performed (Aguirre et al., 2008; Ling et al., 2014a,b). As mentioned already, chemically doped molecules not necessarily resemble their light-induced counterparts, and different EPR-spectroscopic techniques are sensitive to a different extent to these deviations.

## 4. Time-Resolved EPR Spectroscopy of Triplet States

As the main focus of the author's research as well as the remainder of this article is on TREPR spectroscopy of light-induced triplet states, some of their crucial characteristics from an EPR spectroscopist's perspective will be detailed below. The first assignment of triplet states as origin of phosphorescence dates from the middle of the twentieth century (Lewis and Kasha, 1944), and the first recorded EPR spectrum of triplet states was reported some years later (Hutchison and Mangum, 1958, 1961). TREPR spectroscopy following pulsed laser excitation was developed and demonstrated first on a triplet state as well (Kim and Weissman, 1976).

Triplet states consist of two strongly coupled unpaired electrons, with coupling originating from of both, dipolar and exchange interaction between the two electron spins. Whereas the exchange interaction follows an exponential dependence on the distance, the dipolar interaction has an inverse cubed distance dependence. Hence, for triplet states with a rather short distance between the two unpaired spins, the exchange interaction well exceeds the dipolar interaction and completely separates the singlet energy level from the three triplet energy levels. Furthermore, the three triplet energy levels are good eigenstates of the corresponding spin Hamilton operator. As a result, normally, the dipolar interaction entirely dominates the TREPR spectra of light-induced triplet states of organic molecules. Due to symmetry considerations, the three triplet energy sublevels are not equally populated, giving rise to spin polarization and thus greatly enhancing the signal intensities in EPR spectroscopy. See van der Waals and de Groot (1967) for a detailed account on how the symmetries determine the population of the triplet energy sublevels.

**Figure 3:**
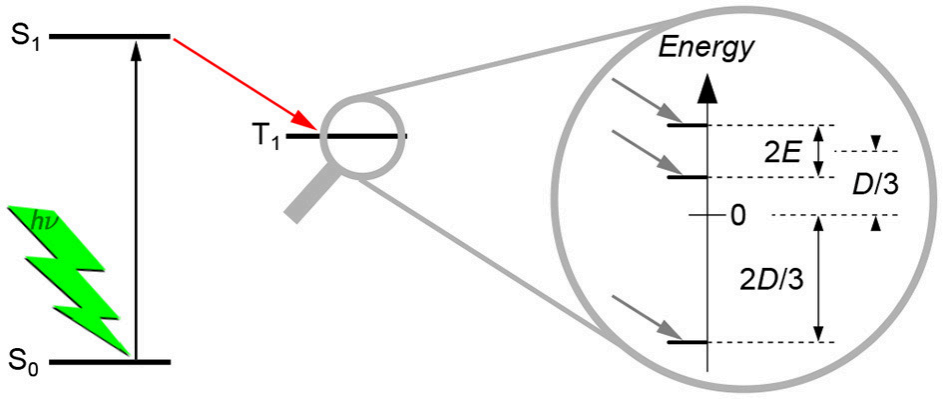
Zero-field splitting of the energy levels of a triplet state. Typically, triplet states are formed via intersystem crossing from the excited singlet state after light excitation. Due to symmetry considerations, the three triplet energy sublevels are not equally populated, giving rise to spin polarization. The splitting of the three triplet energy sublevels without external magnetic field (zero-field splitting, ZFS) is due to the dipolar interaction between the two unpaired electron spins. The splitting can be described by the two scalar parameters *D* and *E*, as shown. For details of their meaning and definition, see the text. Normally, these parameters can directly be estimated from the TREPR spectra of triplet states, cf. Figure 4.

The dipolar interaction gives rise to a splitting of the energy levels even without applied external magnetic field, hence termed zero-field splitting (ZFS), as shown schematically in Figure 3. This splitting can be described by two scalar parameters, *D* and *E*, of the corresponding ZFS tensor **D**. As TREPR spectra of spin-polarized triplet states of organic molecules recorded at X-band frequencies (about 9.5 GHz) and magnetic fields (about 340 mT) are normally dominated by the ZFS interaction, the Hamilton operator H used to describe the system reduces dramatically. The only contributions that need to be taken into account are the Hamilton operator for the electron Zeeman interaction, $H_{\text{EZ}}$, and the one for the ZFS interaction, $H_{\text{ZFS}}$:

(2) $$\begin{array}{rc} H & =H_{\text{EZ}}+H_{\text{ZFS}}=\text{g}\mu_{\text{B}}\text{SB}+\text{S}D\text{S}. \end{array}$$

**Figure 4:**
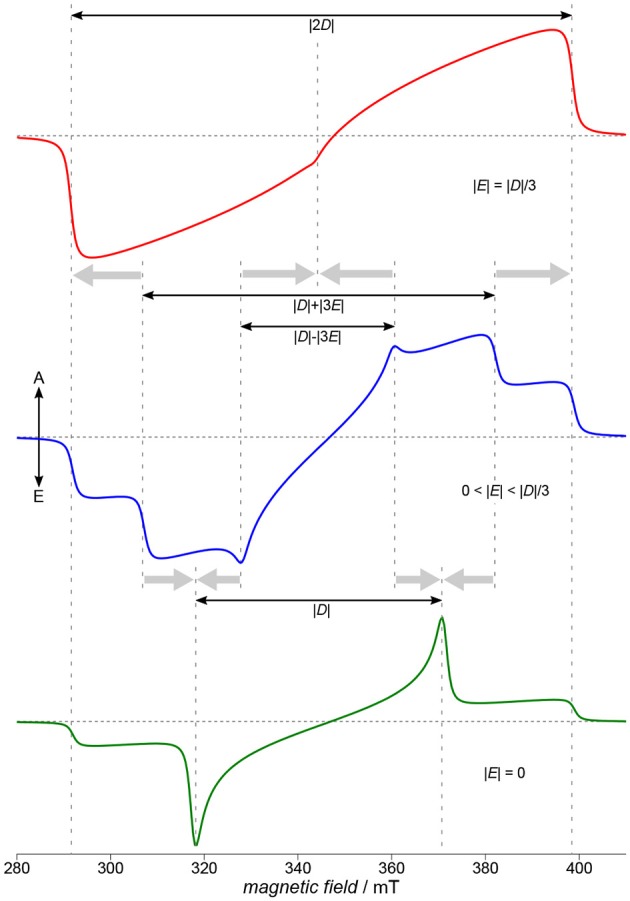
Characteristics of TREPR spectra of (photo-generated) triplet states. Three characteristic situations for the ratio of the two parameters *D* and *E* of the ZFS tensor are depicted here: the fully rhombic case (top, red), an intermediate case (blue, middle) and a fully axial case (green, bottom). Whereas in the intermediate case (blue, middle) all three principal axes of the **D** tensor are clearly visible in the spectrum, this is no longer the case for either fully axial or fully rhombic situations (bottom, green, and red, top, respectively). Whereas assigning *D*_*x*_ and *D*_*y*_ is usually not possible only from the spectra, the *D*_*z*_ position is always assigned to the outermost shoulders of the spectrum. Spectra were calculated using EasySpin (Stoll and Schweiger, 2006). The zero-field populations *p*_1, 2, 3_ of the three triplet sublevels are far from thermal equilibrium, due to optical excitation and the inherent anisotropy of the intersystem crossing processes. Therefore, signals consist of both, absorptive (A) and emissive (E) contributions.

All other contributions, including hyperfine interactions with nearby nuclei, can be considered as small perturbations that can be accounted for using (inhomogeneous) line broadening. The **D** tensor in its principal axis system is given to:

$$\begin{array}{ll} \text{D} & =\left(\begin{array}{ccc} -\frac{1}{3}D+E & 0 & 0\\ 0 & -\frac{1}{3}D-E & 0\\ 0 & 0 & \frac{2}{3}D \end{array}\right), \end{array}$$

where *D* and *E* are the ZFS parameters that can be directly read out from the experimental spectra (cf. Figure 4). They are related to the eigenvalues of the **D** tensor as follows:

$$\begin{array}{ll} D & =\frac{3}{2}D_{z}\;E=\frac{D_{x}-D_{y}}{2}. \end{array}$$

Note that *D* and *E* are usually defined such that the relation |*E*| ≤ |*D*|/3 always holds, resulting in the following convention for the order of the three principal values of the **D** tensor:

$$\begin{array}{ll} \left|D_{z}\right| & >|D_{y}|>|D_{x}|. \end{array}$$

Whereas it is not trivial to directly relate *D* and *E* to the shape of the spin density of a molecule, generally, *D* can be assigned to the average mean distance between the two strongly coupled electron spins of the triplet state, and *E* to the rhombicity, hence deviation from an axial symmetry. Given the ZFS interaction to entirely dominate the TREPR spectra, the absolute values of the parameters *D* and *E* can be directly read out from the experimental data with reasonable accuracy, as shown in Figure 4. Experimentally assigning the signs of *D* and *E* is demanding, and therefore, usually only absolute values are given. Note that there is some ambiguity with respect to the simulation parameters, namely the three triplet sublevel populations and the signs of *D* and *E*, for a given spectral shape. Whereas assigning *D*_*x*_ and *D*_*y*_ is only possible with knowing about the sign of *D* and *E*, due to the convention mentioned above, *D*_*z*_ is always connected to the total spread of the TREPR spectrum of the triplet state. This easy access to the *D*_*z*_ position allows to reveal its orientation in a (partially) oriented sample with respect to the external magnetic field and can hence be used to probe orientation and overall degree of order in, but not restricted to, films of semiconducting polymers on substrates (Biskup et al., 2015).

Depending on the rhombicity, three general cases can be distinguished, as shown schematically in Figure 4. Starting off with the intermediate case (blue, middle), this is the situation for 0 < |*E*| < |*D*|/3 found most often and allowing to extract absolute values for *D* and *E* even graphically from the spectra. Proceeding from this situation either toward the fully axial case with |*E*| = 0 or the fully rhombic case with |*E*| = |*D*|/3, the positions for either *D*_*x*_ and *D*_*y*_ or for all three principal values of the **D** tensor collapse, as shown in Figure 4. As 2|*E*| is defined here as the separation of the energy values of *D*_*x*_ and *D*_*y*_ (cf. Figure 3), in the fully axial case with |*E*| = 0 the two energy levels are degenerate and the respective lines in the spectrum collapse (Figure 4, bottom, green line). The other extreme, the fully rhombic case with |*E*| = |*D*|/3, results in the two innermost lines of the EPR spectrum to fall on top of each other and, due to their opposite polarization, to cancel. The other lines collapse as well, but on the outermost position usually referred to as *D*_*z*_ (Figure 4, top, red line). Fully axial triplet spectra are rare, as they require a particular high symmetry of the excited state. Naphthalenediimide is one notable example of a triplet spectrum that can be ascribed to a fully axial excited state (Meyer et al., 2018b). Sometimes, an increase in rhombicity when proceeding from small building blocks to the polymer can be ascribed to the curvature of the polymer backbone (Matt et al., 2018b).

Normally, EPR spectroscopists, as well as NMR spectroscopists, always seek for higher fields and frequencies to enhance the available spectral resolution. This is necessary, e.g., to resolve the normally quite isotropic **g** tensor of organic molecules. However, for TREPR spectroscopy of (light-induced) triplet states of organic molecules, this is neither necessary nor beneficial, as the dominating interaction in the triplet state is the ZFS that is independent of the magnetic field. Additionally, for conventional X-band EPR spectrometers, the Zeeman splitting introduced by the interaction with the applied magnetic field is larger than the ZFS, hence the latter is already fully resolved. Note that for high-spin states, particularly transition metal ions and alike, this is not usually the case, and here, going to higher fields can indeed be an advantage (McInnes and Collison, 2016; Telser, 2017). Staying with X-band frequencies and fields comes with a number of advantages as compared to high-field EPR. Firstly, sample handling is easier, particularly for films on flat surfaces and alike, as sample dimensions normally scale with the frequency, leading to sample tube diameters for W-band frequencies (≈ 94 GHz) of less than one millimeter. Other advantages include that X-band EPR spectrometers are much more wide-spread and allow for easier light access. Whereas fiber optics can generally be used for high-field EPR spectrometers as well, they are no choice once it comes to polarized excitation necessary, e.g., for magnetophotoselection experiments (Siegel and Judeikis, 1966; Thurnauer and Norris, 1977; Tait et al., 2015a).

Finally, a short note on the time domain of TREPR spectra. Usually, TREPR spectra are recorded at consecutive magnetic field positions, and using a transient recorder, the complete time profile for each magnetic field point is obtained (Weber, 2017). However, extracting relaxation rates from the time profiles is rather involved, as both, transverse and longitudinal relaxation are superimposed in the decay. Additionally, obtaining data in presence of a continuous microwave field further complicates the matter, as its strength usually entirely dominates the time profile. For a more detailed discussion of the different regimes and the proper analysis of TREPR time profiles see Furrer et al. (1980) and Stehlik et al. (1989b). As TREPR spectra of triplet states usually show a rather simple kinetic behavior, mostly an exponential decay, typically, only slices along the magnetic field axis taken at the maximum of the signal and sometimes averaged over a small time window for better signal-to-noise ratio, are shown. It should be noted, however, that neither the signal rise time nor the decay can easily be connected to kinetic parameters obtained from optical measurements. Even in case of strong signals allowing to record time profiles at very low microwave field strengths to obtain relaxation rates, these rates not necessarily reflect the lifetime of the triplet state as such. Often, the decay of non-Boltzmann population of the triplet energy sublevels is faster than the decay of the triplet state as such, and whatever process is faster determines the decay of the EPR signal. Lifetimes of the paramagnetic state as such can be obtained by pulsed EPR experiment, although for triplet states, this usually requires measurements at helium temperatures (10–20 K) due to the fast spin relaxation.

## 5. Triplet States Reveal Insights Into Structure–Function Relationship

**Figure 5:**
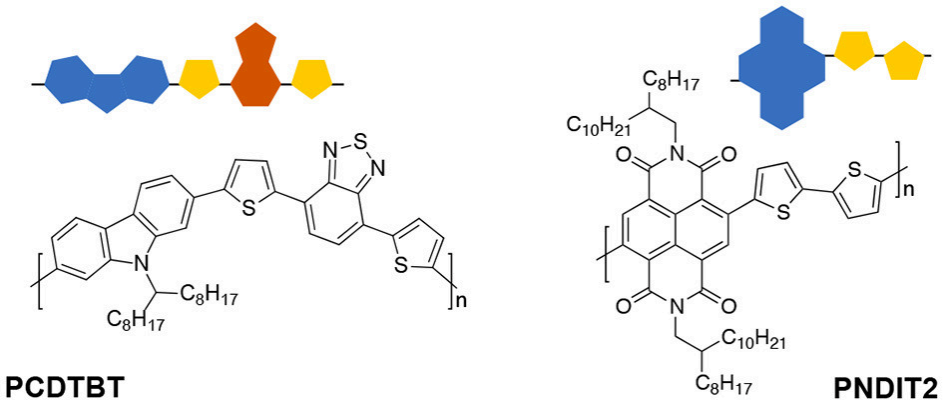
Chemical structures of the two polymers investigated, together with the symbols used in the following. PCDTBT is a p-type polymer known for its rather high efficiency combined with long-term stability. PNDIT2 is a n-type polymer with remarkable charge carrier mobilities.

Two aspects are crucial for device efficiency of OPVs: electronic structure (Scharber et al., 2006) and morphology (Jackson et al., 2015). Investigating light-induced triplet states by TREPR spectroscopy can contribute insights to both aspects, as detailed hereafter. Results obtained on two different polymer systems are presented. For chemical structures and corresponding symbols used, cf. Figure 5. The copolymer poly[*N*-9′-hepta-decanyl-2,7-carbazole-*alt*-5,5-(4′,7′-di-2-thienyl-2′,1′,3′-benzothiadiazole)] (PCDTBT) (Blouin et al., 2007, 2008) is mostly used as donor material in OPVs with high internal quantum efficiency of nearly 100% (Park et al., 2009). Together with its stability under ambient conditions, leading to an increased lifetime of devices of several years (Cho et al., 2010; Peters et al., 2011; Sun et al., 2011; Kong et al., 2014), it is considered a benchmark polymer replacing P3HT in this respect (Beaupré and Leclerc, 2013). Whereas p-type organic semiconductors are paramount, only very few n-type conjugated polymers are known to date, with poly{[*N*,*N*′-bis(2-octyldodecyl)-naphthalene-1,4,5,8-bis(dicarboximide)-2,6-diyl]-*alt*-5,5′-(2,2′-bithiophene)} (PNDIT2), also known as P(NDI2OD-T2) or Polyera ActivInk N2200 (Chen et al., 2009; Yan et al., 2009), being a notable exception. Furthermore, this polymer has a remarkable electron mobility of up to $\mu_{\text{e}}=3\text{c}{\text{m}}^{2}{\left(\text{Vs}\right)}^{-1}$ (Yan et al., 2009; Matsidik et al., 2015), which is correlated with a high degree of molecular order on the micro and nano scale (Caironi et al., 2011; Fazzi et al., 2011).

Morphology of conjugated polymers is a key aspect for device efficiency (Olivier et al., 2014; Jackson et al., 2015), often impairing the theoretically achievable power conversion. Whereas there are many different well-established methods to probe polymer morphology in films (Rivnay et al., 2012), TREPR spectroscopy adds a unique capability probing the overall morphology of a sample combined with molecular resolution due to its sensitivity to the direct surrounding of the electron spin. This can be used to determine both, orientation of the polymer backbone with respect to a surface as well as the overall degree of order in a film (Biskup et al., 2015). Furthermore, TREPR spectroscopy allows to probe polymer morphology in concentrated solution. Both, the choice of solvent and the potential preaggregation have tremendous impact on the final film morphology. Due to the high concentrations used during device fabrication, conventional optical spectroscopy often comes to its limits. The intrinsic high sensitivity of TREPR spectroscopy to the local environment of the (triplet) exciton makes it an excellent tool for investigating polymer morphology under these circumstances (Meyer et al., 2018a).

The degree of (de)localization of excitons in organic semiconductors is of outstanding importance, as it directly relates to efficiency. Depending on the desired application, either localized (OLEDs) or delocalized (OPVs) excitons are sought-after. Due to the direct relationship of the dipolar coupling of the two electron spins in a triplet exciton, TREPR spectroscopy gives a handle on exciton delocalization, revealing striking differences for different polymers (Matt et al., 2018b; Meyer et al., 2018b).

Last but not least, to further investigate the still highly debated role of triplet excitons in OPV devices, spectroscopic methods that allow to probe these states are of outstanding importance. TREPR spectroscopy offers unique capabilities here, allowing not only to directly probe triplet states, but as well to unequivocally assign and discriminate them from other paramagnetic states and to reveal the origin and formation pathways of these excitons (Meyer et al., 2017; Matt et al., 2018a).

### 5.1. Film Morphology: Orientation and Degree of Ordering

The importance of molecular orientation on device efficiency has been discussed in quite some detail in the literature (Rand et al., 2012; Akaike et al., 2015; Ayzner et al., 2015; Kitchen et al., 2015; Osaka et al., 2015). Due to the dipolar coupling of their two electron spins, triplet states are intrinsically sensitive to their orientation toward the external magnetic field. Hence, they can be used to deduce information about the orientation of a molecule with respect to a given reference. In context of organic electronics, where the semiconducting molecules are mostly deposited as thin films on flat substrates, this opens up the possibility to obtain information about the orientation of a molecule with respect to the surface. Given exciton and charge transfer in semiconducting polymers to normally be highly anisotropic, this piece of information can be quite essential to develop efficient devices and control the morphology of the active layer.

There are different methods to obtain the orientation of a molecule in thin film (DeLongchamp et al., 2012; Liu et al., 2013), mostly X-ray scattering methods (Rivnay et al., 2012), but as well transmission electron microscopy (TEM) (Wittmann and Lotz, 1990; Brinkmann, 2011), that are routinely used. TREPR spectroscopy of light-induced triplet states can nicely complement these methods, as it allows to probe rather thick films as used in some devices, and probes normally the entire film, not only the surface.

**Figure 6:**
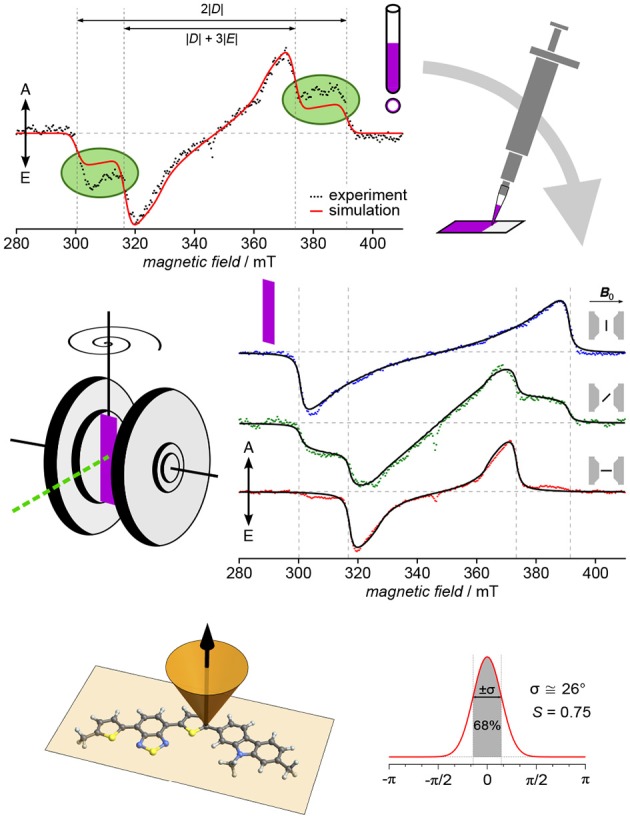
Orientation and degree of ordering of polymer films revealed by angular-dependent TREPR spectroscopy. Particularly in the case of PCDTBT, investigating thin films deposited on the inner wall of an EPR tube resulted in signals that can only fully be described taking into account partial orientation. For a more defined sample morphology and geometry, films were drop-cast on substrate and subjected to angular-dependent TREPR spectroscopy. The resulting spectra reveal rather strong preferential orientation with respect to the surface plane. Using global analysis in conjunction with an intuitive approach to account for the partial orientation by weighting with a Gaussian, both, the orientation of the polymer backbone with respect to the surface (face-on) as well as the overall degree of ordering could be revealed. All TREPR spectra have been recorded at 80 K and X-band frequencies (about 9.7 GHz). For details see Biskup et al. (2015).

The first conjugated polymer for which TREPR spectroscopy of light-induced triplet excitons has been used to determine both, orientation of the polymer backbone toward a surface as well as the degree of ordering, is the highly efficient and air-stable p-type copolymer PCDTBT (Biskup et al., 2015). An overview of the approach as well as the results is given in Figure 6. The original idea was to investigate triplet excitons of PCDTBT originating from intersystem crossing from an excited singlet state in order to be able to distinguish their signature from other signals, e.g., in blends with acceptors. Therefore, a thin film of the polymer was deposited on the inner wall of an EPR tube by evaporating the solvent, as had been done previously (Pasimeni et al., 2001b; Franco et al., 2005; Behrends et al., 2012). Interestingly, though, the first measurements gave clear indication of a preferential orientation, as the outer shoulders of the triplet spectrum (corresponding to the *D*_*z*_ position) could not be fully accounted for using a full powder averaging (Figure 6, top left). To better control sample morphology, films of the pristine polymer were drop-cast onto a flat surface, in this case polyethylene terephthalate (PET), and afterwards, angular-dependent TREPR spectra recorded for different orientations of the sample with respect to the external applied magnetic field. Already on first inspection of the recorded data, a dramatic effect of orientation of the sample can be seen (Figure 6, middle right). From the experimental data, immediately the orientation of the *z* component of the **D** tensor can be deduced, besides the overall strong orientational effect, giving first insight into the orientation of the polymer.

Key aspect of this method to investigate overall orientation and degree of ordering of polymer chains in films on surfaces is the strategy applied to data analysis. Data for all positions of the substrate with respect to the external magnetic field are fitted at the same time with only one set of common parameters for the triplet state, and accounting for the different orientations. Effects of the (partial) orientation are taken into account by weighting the powder average using a Gaussian with two parameters, namely center and width. Using a goniometer to accurately position the sample within the spectrometer, even the relative changes in the angles are known, leaving only an initial offset and the width of the Gaussian as additional fitting parameters, besides those necessary to account for the triplet state. This approach with a Gaussian gives an intuitive handle on both, orientation and degree of ordering of the polymer backbone within the sample. From the width of the Gaussian, the scalar order parameter *S* familiar from liquid crystals (Kuczyński et al., 2002) can be calculated in a straightforward manner.

The rather strong orientation and degree of order in PCDTBT comes quite to a surprise, as this polymer is known to be rather amorphous (Beiley et al., 2011), with only short-range ordering (Lu et al., 2011). At the same time, films of poly(3-hexylthiophene) (P3HT) similarly prepared at the inner wall of an EPR tube show no preferential orientation, although P3HT is known to form semi-crystalline domains of various size (Brinkmann, 2011). Taken together, these results show triplet spectra to be highly sensitive to partial orientation, making them an excellent probe for polymer morphology, complementing the information gained by other methods. TREPR spectroscopy probes whole films, not only surfaces, and gives information on both, orientation and degree of ordering.

Recently, we extended our investigations on the morphology using triplet excitons to other polymers as well. Preliminary data show PTB7 to be strongly oriented as well, similar to PNDIT2 (Meyer, 2017). At the same time, changing from PET substrates to quartz glass plates makes for a more reliable sample placement within the spectrometer, while the stiffness of a quartz glass substrate renders the results more homogeneous, as the flexible PET substrate tends to show a slight curvature within the sample tube. Additionally, this opens up the possibility to investigate the effect of annealing on the polymer morphology, as has been shown for quite a number of different polymers (Lu et al., 2011; Brinkmann et al., 2012). Currently, we are extending our studies on PCDTBT to a series of polymers with varying amount of alkyl side chains. Introducing these side chains has previously been shown to decrease OPV performance, but at the same time to enhance luminescence (Lombeck et al., 2015). Preliminary results show exciton delocalization and overall degree of order in the polymer film to be independent of each other, with the enhanced exciton localization with increasing amount of alkyl side chains to be consistent with the enhanced luminescence of the polymer (Meyer et al., under review; Meyer, 2017). The small but detectable effects on the triplet spectra render the approach valuable, with the additional insight into the overall degree of order in the polymer films to be quite unique.

We note chemically doped films to be a possible alternative to investigate the orientation of polymer films using conventional cw-EPR spectroscopy (Matsumoto et al., 2008). This approach relies on the intrinsic anisotropy of the **g** tensor of the stable radical induced by doping, rather than the anisotropy of the **D** tensor of the light-induced triplet state. Whereas stable radicals allow to use conventional cw-EPR spectroscopy on stable radical species, the rather small **g** anisotropy of organic radicals makes it necessary to measure at high frequencies and fields. The reduced sample dimensions, the small dimension being < 1 mm, make sample preparation rather involved. A very special case is the investigation of the intrinsically paramagnetic copper phthalocyanine (CuPc) (Warner et al., 2012). Here, due to the large **g** anisotropy and the characteristic hyperfine couplings, even conventional cw-EPR spectroscopy at room temperature and X-band frequencies and fields allowed to probe the orientation of the molecule in films with respect to the substrate plane. Note that for assigning the orientation of the molecule, the orientation of the **g** tensor within the molecular frame has to be known, either from quantum-chemical calculations or previous experimental work. However, only a minority of organic semiconductors are intrinsically paramagnetic, rendering this approach less generally applicable.

### 5.2. Solution Morphology: Aggregate Formation and Mode of Delocalization

Another aspect of polymer morphology is the morphology in solution as compared to the situation in films. It has been shown that solution morphology can have a tremendous impact on the final film morphology, both by choice of solvent (Yao et al., 2008; Park et al., 2011; Kim et al., 2016; Xu et al., 2016) and by polymer molecular weight (Koch et al., 2013; Spoltore et al., 2015). Hence, the behavior of polymers in solution is key for the structures and morphologies formed in the solid state. For a wide variety of materials and blends, this has been shown by using high-boiling-point solvent additives such as 1,8-di-iodooctane (DIO) (Machui et al., 2015; van Franeker et al., 2015) that alter the evaporation conditions and therefore the morphology in solution, resulting in entirely different blend morphologies (Rogers et al., 2011; Zusan et al., 2015). Due to using optical spectroscopy, previous studies addressing aggregation of conjugated polymers in solution (Guo et al., 2015; Gross et al., 2017; Panzer et al., 2017) are usually inherently limited to concentrations far below those relevant for device fabrication.

**Figure 7:**
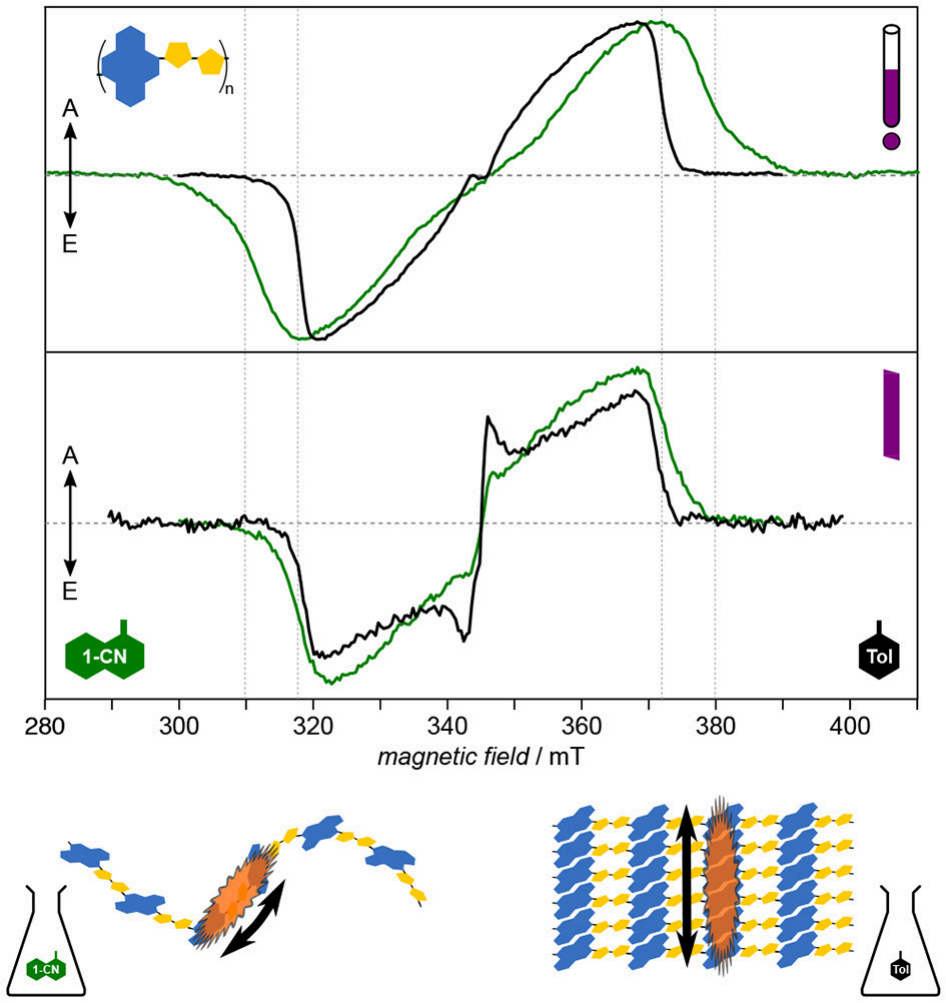
Solution morphology: Solvent-induced aggregate formation in PNDIT2 results in a change in exciton delocalization mode. Already from first inspection, the dramatic difference of the TREPR spectra in both solvents is apparent (upper panel). Careful analysis of the data reveals PNDIT2 to form highly ordered aggregates in toluene (Tol) solution. Additionally, the mode of exciton delocalization changes from intrachain in 1-chloronaphthalene (1-CN) to interchain in toluene (lower panel). Interestingly, the width of the spectra in toluene solution coincide with those obtained for films cast from either solvent (middle panel). This can be explained by PNDIT2 to form aggregates in the film, as known from the literature. All TREPR spectra have been recorded at 80 K and X-band frequencies (about 9.7 GHz). For details see Meyer et al. (2018a).

For PNDIT2, it has been shown previously by optical spectroscopy that the choice of solvent crucially affects the solution morphology, with toluene classifying as “bad” solvent leading to aggregation and 1-chloronaphthalene being a good solvent able to fully dissolve the polymer chains (Steyrleuthner et al., 2012). Owing to its molecular resolution and sensitivity to the direct surroundings of the unpaired electron spin, TREPR spectroscopy provides further detailed insight (Meyer et al., 2018a), cf. Figure 7. Results clearly show aggregation to introduce a high degree of local order in the polymer and to dramatically enhance the delocalization of the exciton. Furthermore, triplet exciton delocalization is only affected by the solvent used and hence by aggregate formation, not by chain length. Finally, aggregation changes the mode of delocalization from intrachain to interchain when forming aggregates, the latter mode dominating as well in thin films. This change in mode of delocalization renders direct comparison of the *D* values obtained from spectral simulations and relating these to the delocalization length rather difficult, as the **D** tensor might reorient with respect to the molecular frame. A similar situation has been observed in chains of porphyrins with increasing length (Tait et al., 2015a).

Assessing the homogeneity of the local molecular environment shows PNDIT2 to occupy a rather large conformational space if dissolved in a good solvent, here 1-chloronaphthalene, in line with theoretical studies (Giussani et al., 2013). This is reflected in the very large inhomogeneous line width as compared to both, toluene solutions of PNDIT2 and other conjugated polymers (P3HT, PCDTBT). It should be noted, though, that the inhomogeneous line width on its own is not a sufficient parameter to reveal aggregation, particularly if compared between different polymer systems. However, comparing one and the same polymer in different solvents and given the dramatic change in spectral shape as observed for PNDIT2, both aggregation as well as the change in mode of delocalization can be clearly assigned. Generally, in terms of their width, spectra for films drop-cast from solution resemble those obtained from toluene solution. However, the inhomogeneous line width of the spectra recorded for films cast from the bad solvent showing aggregation is clearly smaller, attributed to the preformation of aggregates thus leading to a more homogeneous film.

Taken together, TREPR spectroscopy proves to be a valuable tool for investigating aggregation and order in polymers on a molecular length-scale in concentrated solutions relevant for device manufacturing. Morphology of polymers in solution, such as preaggregation effects, has direct impact on the morphology in thin films. Therefore, investigating these effects by TREPR spectroscopy is of particular importance and has direct relation to device efficiency and the optimization of processing conditions. Furthermore, TREPR spectroscopy of light-induced triplet states allows to reveal the mode of delocalization and possible changes, from intra- to interchain. Not only provides TREPR spectroscopy additional information beyond optical spectroscopy, but in addition, it allows to probe concentrations and solvents not readily accessible optically, particularly high concentrations and solvents not forming glasses upon cooling.

### 5.3. Electronic Structure: Exciton Delocalization

Due to its direct relation to efficiency, the degree of (de)localization of excitons in organic semiconductors is of outstanding importance. Depending on the desired application, either localized (OLEDs) or delocalized (OPVs) excitons are in demand. A somewhat simplified picture connects delocalization directly to planarity and conjugation, as the orbital overlap is maximized for parallel orientation of adjacent *p*_*z*_ orbitals (Toutounji and Ratner, 2000). However, deviations from this simple rule in both directions are known, be it strong conjugation despite large torsional angles (Troisi and Shaw, 2016) on the one hand or exciton delocalization confined to a rather small part of the available π system (Tait et al., 2015b) on the other. Eventually, the determining factors for (de)localization are structure and ordering primarily on the molecular scale. This is due to organic semiconductors showing usually rather restricted large-scale ordering in contrast to their inorganic and often highly crystalline counterparts. However, high molecular order as a consequence of a highly panar molecular backbone not necessarily results in higher carrier mobility (Matsidik et al., 2017). Therefore, it is essential to gain a detailed understanding of the electronic structure of polymers and their building blocks in order to develop efficient materials for organic electronics.

TREPR spectroscopy of light-induced triplet excitons allows to access their delocalization length in a unique way, connecting it to both, electronic structure and overall conformational flexibility. To deepen our understanding on the rather complicated relationship between electronic structure, molecular geometry, and exciton delocalization, a systematic experimental approach starting off with the building blocks and proceeding to the polymer, backed up by theoretical calculations, is of high demand. The key aspect of this approach is indeed access to building blocks of different size, as simply extrapolating from the repeat unit is usually neither sufficient nor possible.

**Figure 8:**
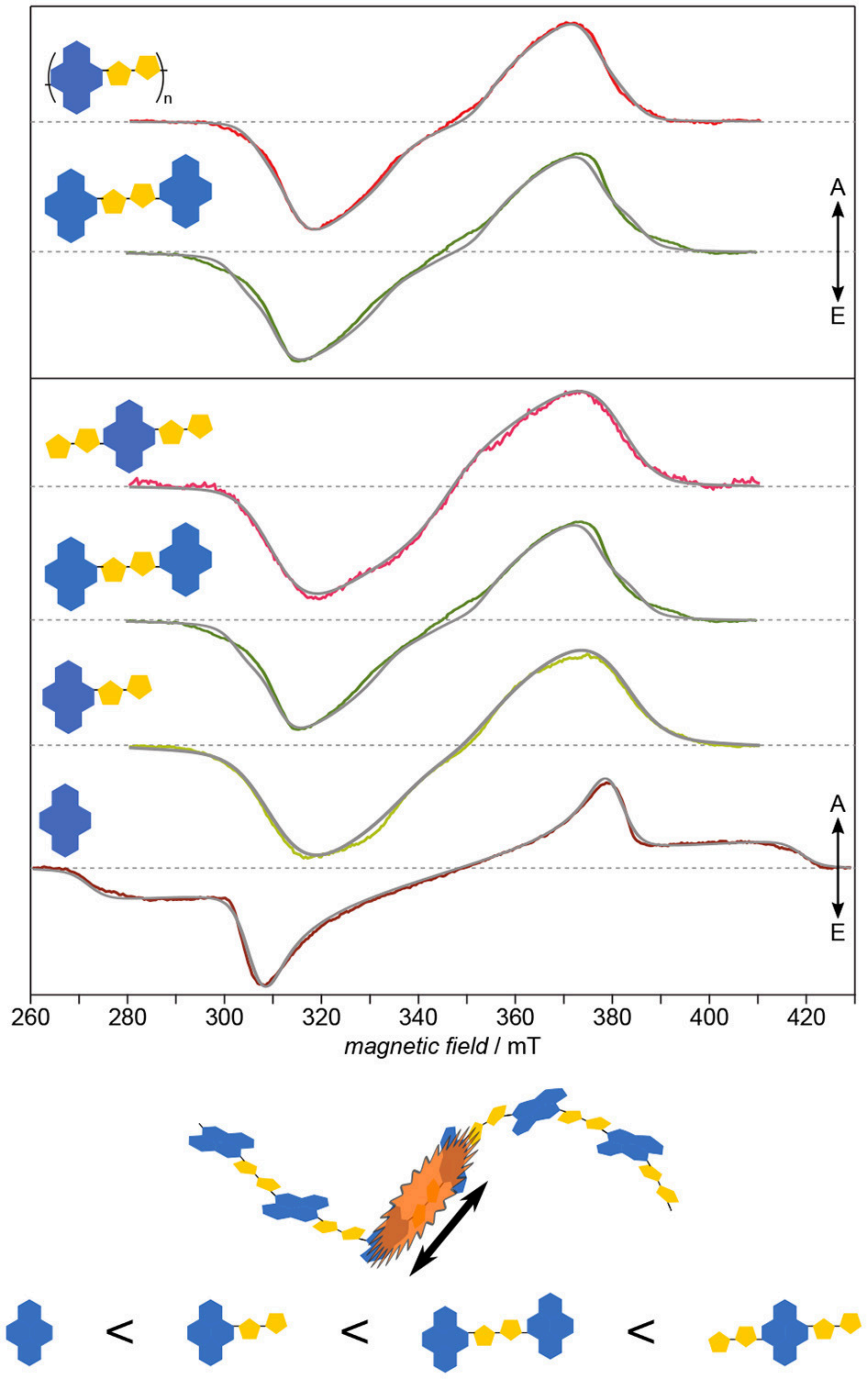
Electronic structure: Exciton delocalization is surprisingly restricted to ≤ 2 repeat units. Systematically investigating PNDIT2 and its building blocks reveals two striking aspects: One of the building blocks shows larger delocalization than the polymer, and another one can serve as model for the latter. Furthermore, the building block exhibiting the spectrum with the largest inhomogeneous line width shows at the same time the largest delocalization. The strong similarity between NDI-T2-NDI and the polymer in terms of their electronic structure leads to assigning the triplet exciton on the polymer to this fragment. All TREPR spectra have been recorded at 80 K and X-band frequencies (about 9.7 GHz). For details see Meyer et al. (2018b).

Applying this approach to PNDIT2 (Meyer et al., 2018b) known for its remarkable charge-carrier mobility quite surprisingly reveals the triplet exciton in this polymer to be strongly confined to no more than two repeat units (cf. Figure 8). This observation explains the lack of correlation between triplet delocalization and inverse chain length shown previously for other polymers such as oligo- and polythiophenes (Bennati et al., 1996; Aguirre et al., 2008) and their derivatives (Steyrleuthner et al., 2017). PNDIT2 is known to be a rather strong push-pull system with the HOMO and LUMO nearly exclusively located on donor and acceptor, respectively (Steyrleuthner et al., 2012). This might explain the unexpectedly strong exciton localization. In any case, these results show that simple extrapolation methods to predict the electronic structure of conjugated polymers from calculations on oligomers, as recently brought forward (Larsen, 2016), are not necessarily universally applicable. In addition to the rather unusual localization, neither planarity of molecules nor the extend of backbone conjugation length seem to be good indicators for the delocalization length of excitons. While optical spectroscopy is mostly restricted to determining relative energy gaps, EPR spectroscopy gives access to the detailed local environment of the molecule, including conformational disorder, and especially the electronic structure. As obvious from both, ZFS parameters and zero-field populations, NDI-T2-NDI is very similar to the polymer and can therefore serve as a model system for the electronic structure of the latter.

Recently, the validity of the approach to use values of |*D*| as measure of the exciton delocalization has been questioned and the strength of hyperfine interactions brought forward as alternative and possibly more reliable alternative (Tait et al., 2015a,b). Generally, the hyperfine interaction should scale linearly with the delocalization. Hence, if the hyperfine interaction of the repeat unit is known either from experiment or quantum-chemical calculations, it can readily be used to quite accurately determine delocalization lengths, as has been shown not only for light-induced triplet states, but as well for doped polymers (Aguirre et al., 2008). However, if the hyperfine interactions are partly due to flexible side chains bearing some spin density, the situation may become much more involved (Steyrleuthner et al., 2017). Most probably, simply relying on either approach will not automatically yield correct results. As long as the electronic structure of the triplet states under investigation is sufficiently similar for the building blocks of increasing length and does not change dramatically, as in the porphyrin case (Tait et al., 2015a), relying on |*D*| for semiquantitative analysis should be fairly accurate. Additionally it is worth mentioning that recording electron-nuclear double-resonance (ENDOR) spectra to obtain the hyperfine couplings of the triplet states, due to their fast spin relaxation, normally requires rather low temperatures (10–20 K). This is only possible by using liquid helium as coolant, not necessarily readily available, as compared to liquid nitrogen, not to mention the additional equipment necessary for this type of double-resonance experiment. ENDOR spectroscopy can, in very simple terms, be considered as an NMR experiment using the electron spin as a probe to increase sensitivity. For details, the interested reader is referred to the literature (Harmer, 2016).

Another aspect of investigating the building blocks of PNDIT2 deserves to be highlighted, namely the observed high conformational flexibility, as apparent from the unusually large inhomogeneous line widths of the TREPR spectra. Its seeming correlation with the delocalization length makes it an interesting candidate to enhance both, intra-chain exciton delocalization and possibly exciton and charge carrier mobility.

Taken together, TREPR spectroscopy of light-induced triplet excitons of a series of building blocks with increasing length provides information beyond exciton delocalization, as the populations of the triplet energy sublevels are highly sensitive to the local molecular environment. Furthermore, showing that planarity not necessarily dictates delocalization length and that high intrinsic conformational flexibility may well be a favourable characteristic of conjugated polymers has some impact on the rational design of conjugated polymers for organic electronics applications. Additionally, electronic structure appears to be of high importance for exciton delocalization and charge-carrier mobility.

### 5.4. Electronic Structure: Identifying the Dominating Building Block

**Figure 9:**
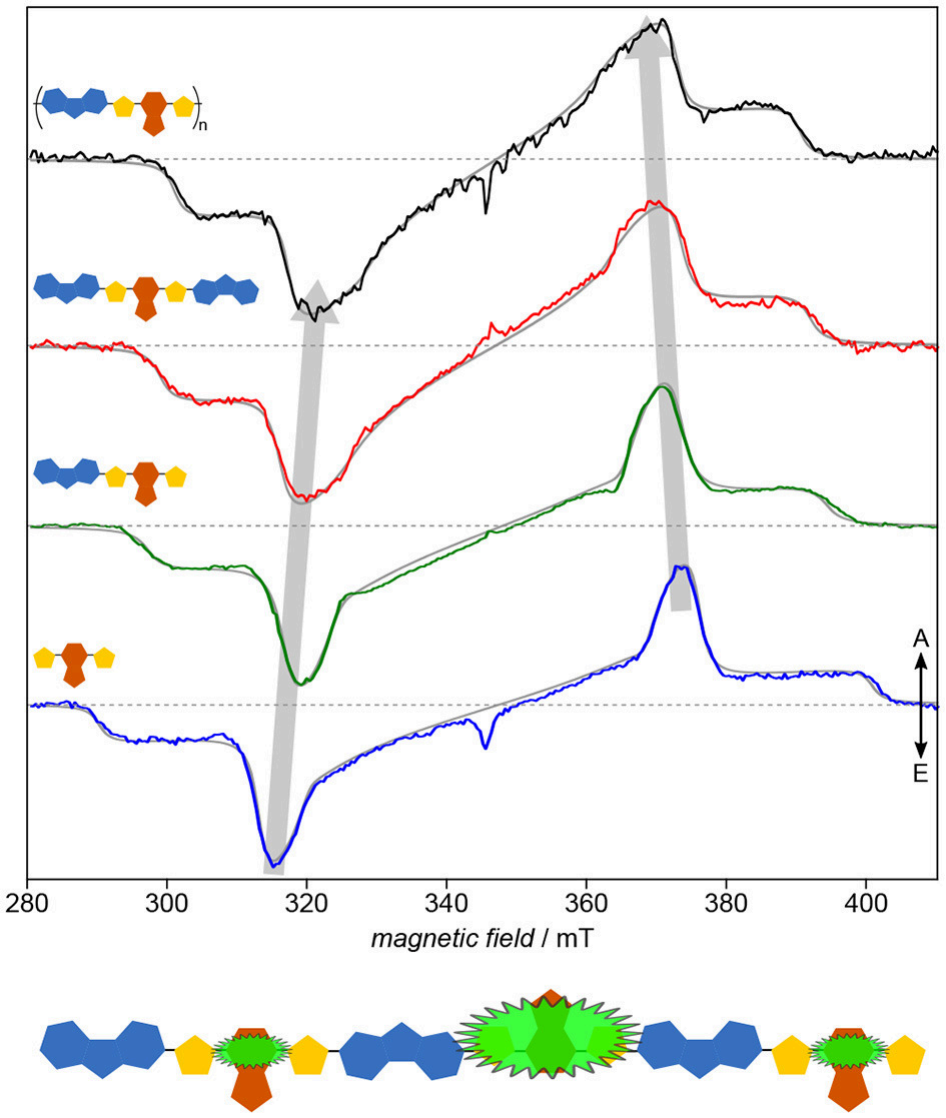
Electronic structure: Identifying the dominating building block. On first sight, all that seems to change when going from the TBT moiety to the polymer PCDTBT is the width of the spectra, being a measure of the exciton delocalization. Both, detailed quantum-chemical investigations as well as spectral simulations of the TREPR spectra (gray solid lines) support this notion. Hence, the TBT unit, in itself a push-pull system, seems to entirely dominate the electronic structure even of the polymer. All TREPR spectra have been recorded at 80 K and X-band frequencies (about 9.7 GHz). For details see Matt et al. (2018b).

As has been shown already in the previous section, systematically investigating building blocks of increasing length and comparing the results with the polymer deepens our understanding of the structure–function relationship in organic semiconductors by means of revealing even subtle details of their electronic structure. Applying this approach to the copolymer PCDTBT (Matt et al., 2018b) known for its efficiency and device stability demonstrates the versatility of the results, particularly if compared to the study of PNDIT2 (Meyer et al., 2018b). Already the inherent similarity of the TREPR spectra of the light-induced triplet states (Figure 9) makes it obvious that the electronic structure of the polymer as well as each of the smaller building blocks is dominated entirely by the TBT moiety. Hence, the usual description of PCDTBT as a carbazole derivative, while chemically entirely correct, is somewhat misleading. Given the widespread use of the TBT unit in other conjugated polymers as well, further investigations into the dominant character of this building block promise to be quite interesting.

The combination of optical and TREPR spectroscopy with detailed quantum-chemical calculations of the spin density reveal the delocalization to extend along the backbone, over at least two repeat units, consistent for singlet and triplet excitons, quite in contrast to other push-pull systems previously investigated, such as PNDIT2. Interestingly, the positive polaron in the polymer has been shown to be delocalized as well over only three repeat units (Niklas et al., 2013), comparable to the triplet exciton. For other polymers, the polaron is much farther delocalized than the triplet exciton (Bennati et al., 1996; Aguirre et al., 2008). PCDTBT as well as all its building blocks show a remarkable homogeneity reflected in the vanishing inhomogeneous line width of their triplet spectra. By ruling out aggregation phenomena (Wang et al., 2014), this is ascribed to a rather rigid and planar backbone geometry. Hence, the increasing rhombicity with longer backbone can directly be ascribed to the intrinsic curvature of the polymer backbone (Risko et al., 2011).

In summary and combined with the results from the investigation of PNDIT2 and its building blocks (Meyer et al., 2018b), these results show the power of TREPR spectroscopy to reveal detailed insight into the electronic structure as well as the local environment with molecular resolution. Again, combining synthetic chemistry for systematic access to building blocks of different size, optical and TREPR spectroscopy, and DFT calculations is crucial to provide detailed insight extending previous detailed optical investigations (Banerji et al., 2012). Particularly the TBT moiety to entirely dominate the electronic structure has potential high impact for other conjugated polymers used in OPV devices, given its widespread use as building block.

### 5.5. Triplet Routes: Spin-Forbidden Direct S_0_→T Excitation

The role of triplet states in organic electronics is still highly debated (Köhler and Bässler, 2009). At least in context of OPVs they are usually regarded to be detrimental for the overall device efficiency (Rao et al., 2013). The energy of the low-lying triplet states of the donor is normally too far below the LUMO level of the acceptor for these triplet states to contribute substantially to charge separation. On the other hand, singlet fission (Smith and Michl, 2010) has been brought forward to push efficiency by generating more than one charge carrier per incident photon, thus overcoming the Shockley–Queisser limit (Shockley and Queisser, 1961). Ideed, already in the 1970s, singlet fission has been observed in tetracence crystals at room-temperature using EPR spectroscopy (Yarmus et al., 1972), and recently, soluble derivatives with relevance for OPV devices have been characterized in more detail (Weiss et al., 2017). The two big advantages of TREPR spectroscopy over optical methods in this context are its direct access to the triplet state as well as its capability to unequivocally assign the paramagnetic species to be a triplet state. But not only can the triplet states as such be assigned. Due to the inherent sensitivity of the triplet sublevel populations to both, the molecular geometry as well as the triplet precursor state, TREPR spectroscopy can even give insight into triplet formation pathways. The most prominent example is triplet states occuring from back electron transfer from a charge-separated state (process 5 in Figure 2 and process 2 in **Figure 11**) and first observed in photosynthetic reaction centers (Thurnauer et al., 1975; Budil and Thurnauer, 1991). The polarization patterns observed for these triplet excitons are quite unique and cannot be generated by intersystem crossing.

**Figure 10:**
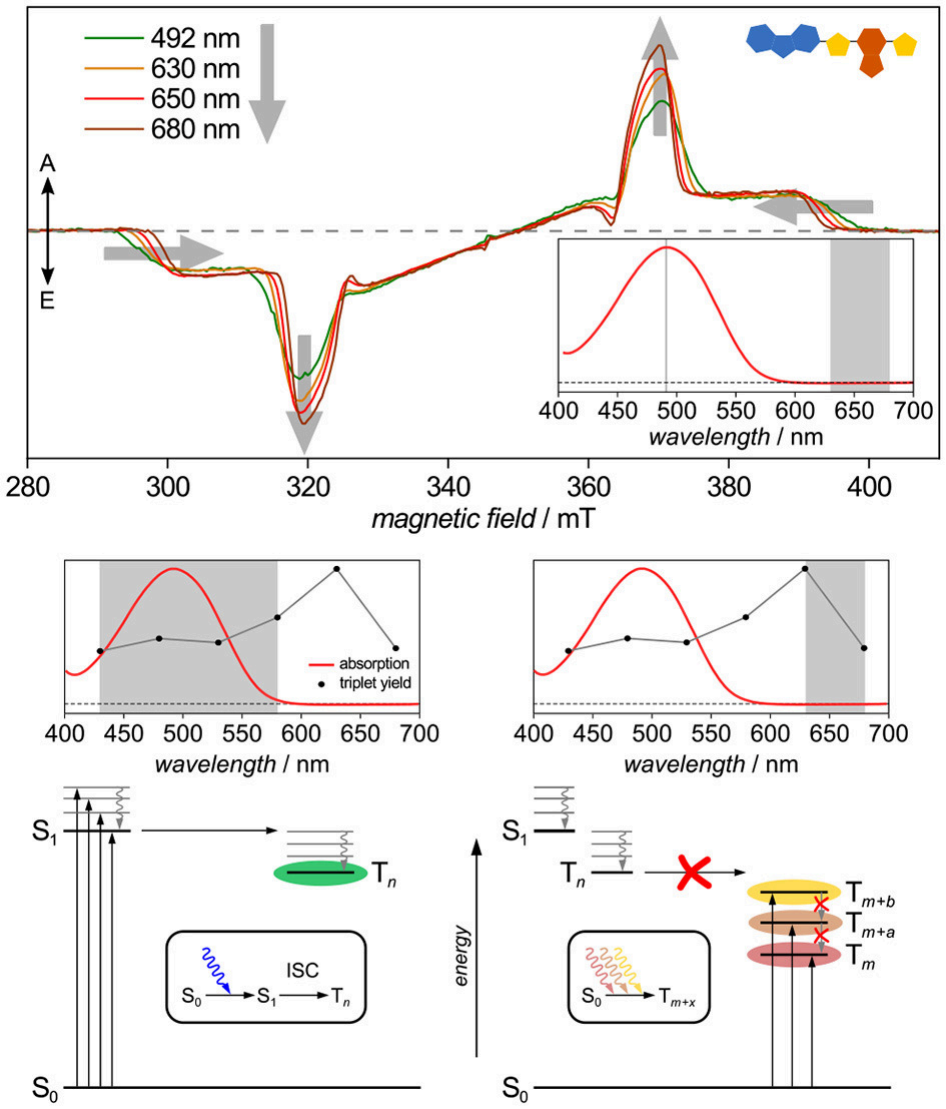
Triplet routes: Evidence for spin-forbidden direct S_0_ → T excitation. Exciting CbzTBT, the repeat unit of PCDTBT, up to 80 nm red-shifted from the onset of its absorption band leads to distinct triplet states that slightly differ in both, shape and intensity from those observed upon excitation within the absorption band and attributed to originating from intersystem crossing from the excited singlet state. Ruling out aggregation and different conformations, this is attributed to a spin-forbidden direct S_0_ → T excitation. All TREPR spectra have been recorded at 80 K and X-band frequencies (about 9.7 GHz). For details see Meyer et al. (2017).

Another potential route toward triplet states is the spin-forbidden direct optical S_0_ → T excitation first demonstrated decades ago by Kasha and others (Lewis and Kasha, 1945; Kasha, 1950; Evans, 1957; Goodman and Laurenzi, 1968). In light of triplet–triplet annihilation processes discussed as potentially boosting efficiency of OPV devices (Gehrig et al., 2015; Yanai and Kimizuka, 2016), this additional route toward triplets may well be relevant if it existed in organic semiconductors. Recently, this pathway has been shown to exist for CbzTBT, the repeat unit of PCDTBT (Meyer et al., 2017), cf. Figure 10. Besides showing distinct triplet states when excited up to 80 nm red-shifted from the onset of the absorption band, in this particular molecule the relative triplet yield seems even to increase when directly exciting into the triplet manifold. Careful analysis and temperature-dependent optical absorption data helped to rule out aggregation to play a role here. Furthermore, alternative explanations, such as different conformations, as recently shown for porphyrins using wavelength-dependent TREPR spectroscopy (Tait et al., 2016), can be ruled out in this case due to the tremendous red-shift in wavelengths.

The relative quantification of triplet yields as shown here for CbzTBT demands a comment. Normally, TREPR spectra cannot be analyzed in a quantitative fashion, as signal intensities strongly depend on a number of experimental and device parameters such as laser adjustment and actual sample position. On an even more fundamental level, these spectra consist of overlapping contributions of differently polarized lines that partially cancel out each other, making the resulting signals particularly sensitive to even slight changes in the electronic structure of the investigated system. In the particular study discussed here (Meyer et al., 2017), special precautions have been taken and the identical sample has been measured under entirely identical conditions with only the excitation wavelength changing. In addition, in this particular case the polarization pattern doesn't change dramatically, rendering a direct comparison possible. Note, however, that no quantification of the triplet yield in terms of quantum efficiencies can be drawn from TREPR data in general.

**Figure 11:**
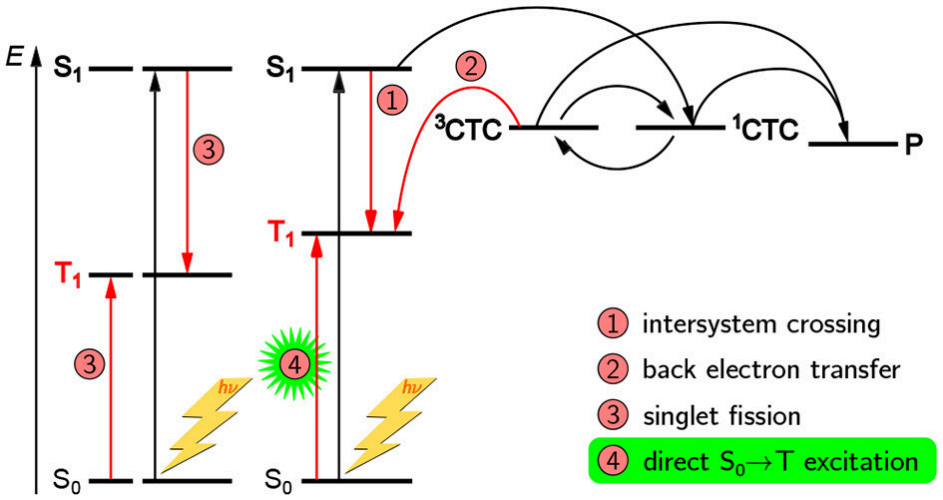
Possible origins of triplet states in OPV devices after light excitation. In an ideal device exhibiting the relative position of the energy levels of the states as shown in addition to very fast charge separation, no triplet states would be created at all. In reality, however, triplet states are unavoidable. Hence, detailed knowledge of their characteristics and possible pathways are highly important. Furthermore, triplet states could be used to boost efficiency, in case of singlet fission leading to internal quantum efficiencies of >1. TREPR spectroscopy, by directly probing triplet states, allows for unequivocally assigning the spin multiplicity as well as shedding light on precursor states.

Low-lying triplet states directly populated via optical (spin-forbidden) transition from the singlet ground state extend and complete the existing picture of pathways leading to triplet states (cf. Figure 11). In polymers, long-wavelength radiation (e.g., near-IR) as available from ambient sunlight could well result in unproductive, energetically low-lying triplet states inaccessible for charge separation and hence contributing to the overall loss of efficiency of OPV devices. However, using acceptors with orbitals energetically matching those of these low-lying triplet states could help boosting the quantum efficiency of the devices by using long-wavelength irradiation for productive charge separation. Additionally, one could make use of pathways to repopulate the singlet state, either thermally (Uoyama et al., 2012; Lee et al., 2016) or by triplet—triplet annihilation (Gehrig et al., 2015). Very recently, we could show by investigating the TBT moiety alone that this unit seems largely responsible for the observed direct S_0_ → T optical excitation (Matt et al., 2018a).

Taken together, these results demonstrate the unique insight from TREPR spectroscopy that cannot be obtained by other spectroscopic methods. Particularly the direct access to triplet states and the possibility to identify the observed spectra with a chemical species render TREPR spectroscopy superior to optical methods for investigating triplet states. In contrast, optical spectroscopy, particularly with long path lengths, suffers strongly from potential impurities in the solvent and the lack of direct assignment of the absorption bands to a distinct molecule, besides that it cannot detect the spin multiplicity of an observed species. Additionally, certain pathways lead to very distinct populations of the triplet sublevels reflected in characteristic polarization patterns that can unequivocally be assigned, such as triplet states formed via back electron transfer (Thurnauer et al., 1975; Budil and Thurnauer, 1991). Those states have been observed and assigned in OPV materials as well (Niklas et al., 2015), allowing even to disentangle the contributions due to several different and concurrent triplet formation pathways (Thomson et al., 2017).

## 6. Summary: Accessible Aspects of Structure–Function Relationship

**Figure 12:**
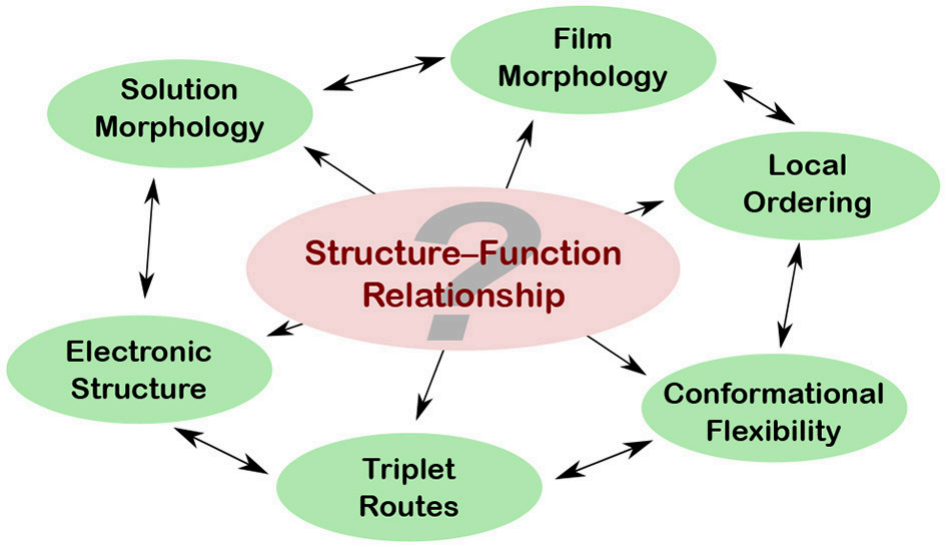
Aspects of structure–function relationship of OPV materials accessible by TREPR-spectroscopic investigations of light-induced triplet states. Whereas the list is by no means complete, for all aspects listed here TREPR spectroscopy has been shown to contribute valuable insight. EPR spectroscopy in general, due to its broader context and applicability, will clearly help to further deepen our understanding of fundamental aspects and mechanisms of OPV materials and devices.

As has been demonstrated above, EPR spectroscopy, and particularly its time-resolved variant TREPR, can contribute crucial insight into structure–function relationship of organic semiconductors with direct relevance for improving device efficiency, complementing results obtained using other methods. An overview of the aspects of structure–function relationship that have been shown so far to be accessible by TREPR-spectroscopic investigation of light-induced triplet states is given in Figure 12.

As TREPR spectra of light-induced triplet states are highly sensitive to partial orientation, TREPR spectroscopy is highly suited to probe film morphology, revealing information on both, orientation and degree of ordering. However, investigations are not limited to films. With its sensitivity to the direct molecular environment of the paramagnetic species, TREPR spectroscopy allows to probe the solution morphology of conjugated polymers in high concentrations necessary for device manufacturing that are normally not accessible by optical spectroscopy. Here, not only aggregation, but degree of local ordering as well as the mode of exciton delocalization (intra- vs. interchain) can be accessed. Both, conformational flexibility as well as local ordering are reflected in the inhomogeneous line width of the TREPR spectra obtained. Generally, the better solvated a polymer chain, the higher its intrinsic flexibility. In contrast, aggregates tend to create highly ordered local environments. Information obtained on the electronic structure of the materials reaches far beyond exciton delocalization, as the triplet populations are highly sensitive to the local environment. Hence, not only the unusual restriction of the triplet exciton delocalization in PNDIT2 could be successfully revealed, but as well the dominant role of the TBT moiety in PCDTBT not readily accessible by other spectroscopic means, but fairly consistent with quantum-chemical calculations that could thus be validated. Eventually, due to its exclusive sensitivity to paramagnetic states and the capability to directly probe and unequivocally assign triplet states, routes toward triplet excitons can be revealed. Here, the populations of the three triplet energy levels can reveal the underlying ISC mechanism. This not only allows to identify triplet states originating from back electron transfer due to their characteristic and unique polarization pattern, but revealed direct optical S_0_ → T excitation to exist in organic semiconducting materials.

Beyond this wealth of information accessible via investigating light-induced triplet states, TREPR of charge-transfer complexes as well as conventional and light-induced EPR can be used to investigate even further aspects of structure–function relationship in OPV materials and eventually relate the molecular characteristics on the microscopic scale with the macroscopic parameters such as device efficiency.

## 7. Conclusions

With its unique access to paramagnetic states ubiquitous in OPV devices, EPR spectroscopy is an excellent tool to shed light on the fundamental questions of the mechanisms underlying OPV device operation. Furthermore, it complements nicely existing and well-established methods to characterize the materials. Using light-induced triplet states as local probes with molecular resolution has proven to be particulary suited to investigate the structure–function relationship of conjugated polymers used in organic electronics and OPVs, ranging from film and solution morphology to insights into the electronic structure and triplet formation pathways. Indispensable for the success but is its combination with synthetic chemistry, providing not only the polymers, but as well building blocks of different length, as well as with other spectroscopic and quantum-chemical methods. Extending the approaches laid out here to other polymer systems will clearly deepen our understanding of these materials and eventually provide the necessary information to further improve device efficiency and stability.

## Author Contributions

The author confirms being the sole contributor of this work and has approved it for publication.

### Conflict of Interest Statement

The author declares that the research was conducted in the absence of any commercial or financial relationships that could be construed as a potential conflict of interest.
